# Mobile Health Intervention Tools Promoting HIV Pre-Exposure Prophylaxis Among Adolescent Girls and Young Women in Sub-Saharan Africa: Scoping Review

**DOI:** 10.2196/60819

**Published:** 2025-06-20

**Authors:** Alex Emilio Fischer, Homaira Hanif, Jacob B Stocks, Aimee E Rochelle, Karen Dominguez, Eliana Gabriela Armora Langoni, H Luz McNaughton Reyes, Gustavo F Doncel, Kathryn E Muessig

**Affiliations:** 1 College of Nursing Florida State University Tallahassee, FL United States; 2 Institute on Digital Health and Innovation Florida State University Tallahassee, FL United States; 3 CONRAD Eastern Virginia Medical School Macon & Joan Brock Virginia Health Sciences at Old Dominion University Norfolk, VA United States; 4 Department of Health Behavior Gillings School of Global Public Health University of North Carolina at Chapel Hill Chapel Hill, NC United States

**Keywords:** HIV, pre-exposure prophylaxis, adolescent girls and young women, mHealth, app, intervention

## Abstract

**Background:**

In 2022, 3100 adolescent girls and young women in sub-Saharan Africa experienced new HIV infections each week. HIV pre-exposure prophylaxis (PrEP) is effective at preventing HIV but has limited uptake and persistence. Mobile health (mHealth) interventions can improve medication adherence; however, their utility to improve PrEP adherence among adolescent girls and young women is not well established.

**Objective:**

This scoping review synthesizes evidence supporting mHealth for PrEP among adolescent girls and young women in sub-Saharan Africa and identifies strategies for further evaluation.

**Methods:**

We searched PubMed and Google Scholar databases, expert referrals, and reference lists using the following eligibility criteria: (1) original research study or protocol; (2) English language; (3) publication between January 1, 2012, and August 31, 2023; (4) inclusion of adolescent girls and young women; (5) conducted in sub-Saharan Africa; and (6) use of mHealth tools to promote PrEP uptake, adherence, or persistence. Titles and abstracts were screened by 2 independent researchers. Full-text manuscripts were reviewed against all eligibility criteria to determine the final included studies. The characteristics and results of the included studies were abstracted and synthesized by mHealth tool type.

**Results:**

The search identified 482 unique citations. Title and abstract review removed 380 citations primarily for not including adolescent girls and young women or being conducted outside sub-Saharan Africa. The remaining 102 articles underwent full-text review, yielding 31 eligible publications reporting on 21 unique studies. The most common mHealth tool was SMS text message (n=11), followed by app (n=9), telehealth (n=3), website (n=4), and video (n=1). Few publications evaluated effectiveness, and the results were mixed. One study found that SMS text message reminders improved PrEP adherence, and another concluded that SMS text message reminders did not show a significant impact. Two studies found that differentiated service delivery, which included mHealth components, improved PrEP uptake or persistence; however, the findings could not be attributed solely to the mHealth components. Lastly, 1 website was shown to improve PrEP persistence. Several earlier-stage studies focused on values and preferences toward mHealth without reporting the impact on PrEP.

**Conclusions:**

We found few rigorously evaluated mHealth interventions for supporting PrEP among adolescent girls and young women, preventing the ability to draw conclusions on its effectiveness. Studies documented high usability and acceptability but limited assessment of the impact on health outcomes. Secondary uses of mHealth were found for data collection and components of the standard of care. There is substantial room for growth in the innovative use of mHealth to support PrEP among adolescent girls and young women. Consideration of the strengths and limitations of mHealth tools in the local setting, review of past lessons learned, and intentional measurement of mHealth exposure and use could help advance this growing field.

## Introduction

### Background

Since 2010, new global HIV infections have decreased by 39%, but the occurrence of 1.3 million new infections in 2022 still exceeds the 2020 Joint United Nations Programme on HIV and AIDS (UNAIDS) target of 500,000 [1]. The decrease in new infections coincides with the global scale-up of preventative services in high-prevalence countries; however, more work is needed to reach the ambitious target of 370,000 new infections by 2025, especially among key populations [2,3]. Over half of all new HIV infections are in sub-Saharan Africa, and 62% of those are among adolescent girls and young women [1].

Adolescent girls and young women in sub-Saharan Africa experience factors across multiple levels that increase their likelihood of acquiring HIV, including biological, socioeconomic, cultural, and system-level factors [4,5]. Further, economic vulnerability in this population contributes to power imbalance in relationships, influences partnerships and condom use, and increases the likelihood of exposure to HIV through age-disparate relationships and transactional sex [4,6,7].

When HIV pre-exposure prophylaxis (PrEP) is taken consistently as a daily oral pill, it is effective at preventing HIV infections among men who have sex with men, serodiscordant couples, and people who inject drugs; however, significant differences in effectiveness exist across geographic locations and populations [8-10]. Women have had less robust prevention results, with inconsistent adherence and discontinuation contributing to these outcomes, alongside biological differences that require higher adherence to reach protective drug levels in vaginal tissue [9,11,12].

Although PrEP uptake among adolescent girls and young women in sub-Saharan Africa continues to increase, few countries have a PrEP-to-need ratio over 1 (having more individuals on PrEP than new HIV infections) [13]. Barriers to PrEP use (uptake, adherence, and persistence) for adolescent girls and young women in sub-Saharan Africa include side effects, stigma, gender-based violence, lack of social support, low risk perception, medical distrust, and the need to regularly access clinics for prescriptions and refills [14-17]. Health systems also face financial and capacity challenges with the introduction of PrEP into an already stressed system [18,19].

Mobile health (mHealth) tools have the potential to mitigate a number of individual-level barriers to PrEP use, without adding substantial strain to health care workers and systems [20,21]. A broad range of mHealth interventions have been shown to increase PrEP adherence among sexual and gender minority men in high-income countries [22,23] and to increase HIV antiretroviral therapy (ART) adherence among adolescent girls and young women in sub-Saharan Africa [24]. mHealth interventions could also be adopted to support women in sub-Saharan Africa to uptake, adhere to, and sustain PrEP, and a detailed understanding of the state of the field could guide the expansion of this work.

### Previous Reviews

We identified 3 recent reviews on general PrEP promotion in low- and middle-income countries (LMICs) [18,19,25]. A 2021 synthesis by Irungu et al [18] (study publication years unspecified) reviewed multilevel interventions for PrEP use among adolescent girls and young women. A 2022 scoping review by Ekwunife et al [19] focused on LMICs and reviewed 11 publications (years 2010 to 2021) that investigated 6 interventions to increase PrEP uptake and continuation among adolescent girls and young women engaged in sex work or transactional sex. Lastly, a 2022 systematic review by Goldstein et al [25] (publication years 2000 to 2021) investigated the use of mHealth interventions for adolescents and young adults (irrespective of gender) in LMICs across the entire HIV cascade. Among 9 identified studies for PrEP adherence, 5 were among adolescent girls and young women in sub-Saharan Africa [25].

Collectively, these reviews identified 9 unique interventions conducted in sub-Saharan Africa that included adolescent girls and young women, and 6 of these had some mHealth component. Four interventions with mHealth tools for adolescent girls and young women in sub-Saharan Africa were common to all reviews: 3 involving SMS text message reminders (HPTN 082, Monitoring PrEP among Young Adult Women [MPYA], and Prevention Options for Women Evaluation Research [POWER]) and 1 involving a phone call to deliver drug-level feedback (3Ps [Perception, Partners, Pills] for Prevention). One review [18] also included an ongoing study comparing SMS text message reminders to WhatsApp support groups (PrEP Sequential Multiple Assignment Randomized Trial [SMART]) and 1 [25] included Mobile Solutions for Women’s and Children’s Health (mWACh), a 2-way SMS text message system for PrEP adherence and continuation support among postpartum women. Overall, the reviews found limited data on the effectiveness of current interventions (including mHealth tools) to increase PrEP uptake and adherence among adolescent girls and young women in LMICs [18,19], and the strength of evidence was limited as most publications were uncontrolled single-arm studies with small sample sizes [25]. Overall, mHealth tools were deemed acceptable and feasible. No studies associated the use of mHealth tools with improved PrEP uptake. Moreover, 1 intervention (POWER) suggested improvement in clinic visit attendance, 2 interventions (POWER and mWACh) found improvements in PrEP continuation, and none found associations with PrEP adherence. The barriers identified were smartphone access issues, fatigue with SMS text messaging, and the cost of phone data [18].

Our review expands prior work by focusing on the specific geographic context of sub-Saharan Africa and examining mHealth intervention types and components for adolescent girls and young women in greater detail. Many of the studies identified in prior reviews were in pilot and acceptability phases or early trial implementation, and additional impact results and new formative studies have become available over the past few years.

To the best of our knowledge, no prior reviews have focused specifically on mHealth for PrEP among adolescent girls and young women in sub-Saharan Africa, which is the population most affected by the ongoing HIV epidemic. This article aims to address this gap through a scoping review of the literature. Scoping reviews are generally more exploratory than systematic reviews and are designed to synthesize the current evidence and gaps surrounding a particular topic. By describing the current landscape and body of evidence, this review aims to identify promising strategies and key gaps, which can direct future research toward the development and implementation of interventions and the evaluation of new mHealth modalities for supporting HIV prevention in adolescent girls and young women.

## Methods

This scoping review followed a common methodological framework for scoping studies [26] and was modeled on the PRISMA-ScR (Preferred Reporting Items for Systematic Reviews and Meta-Analyses extension for Scoping Reviews) guidelines [27]; however, no review protocol was registered or published.

The review included English language articles that featured one or more mHealth tools to facilitate HIV PrEP, published from January 1, 2012 (the first year a randomized controlled trial [RCT] showed that PrEP could effectively prevent HIV in heterosexual men and women in Africa [28]) until August 31, 2023, when the article search was completed. Articles were eligible if the study population (or a well-defined subpopulation) included women between 14 and 25 years of age in sub-Saharan Africa. Articles were ineligible if the digital tools were developed exclusively for use by health care workers (versus for the PrEP end user) or if the tool was used primarily for research or data collection (versus with the goal of improving end-user PrEP outcomes).

Searches for eligible articles were conducted on PubMed and Google Scholar, with the reference lists of all eligible literature subsequently searched to identify additional articles. Reviews, perspectives, and policy articles were not included as our aim was to synthesize completed and ongoing original research. Grey literature was searched via expert referrals and conference proceedings, so that formative research, pilot studies, and protocol publications could be included for a holistic review. Protocol articles were included as they describe original research that is planned or underway, to capture the directions the field is heading. This information is particularly relevant for encouraging innovative ideas to address current research gaps. While these publications do not directly provide findings on the usability or effectiveness of mHealth tools, they provide important information on the state of the science and future directions in the field.

Advanced search features were used to query the abovementioned databases for groups of keywords encompassing mHealth, adolescent girls and young women, and PrEP. SMS text message, text, mHealth, digital, WhatsApp, smartphone, app, telehealth, and multimedia messaging service (MMS) were used to denote mHealth; women, girls, and adolescents were used to denote adolescent girls and young women; and PrEP and pre-exposure prophylaxis were used to denote PrEP. All articles were recorded in a shared spreadsheet, and then, duplicate articles were removed. The remaining articles were independently screened at the title and abstract levels by 2 co-authors (KEM and AEF), and articles that did not meet the eligibility criteria cited above were subsequently removed. Two co-authors (HH and HLMR) reviewed the list of eligible articles after title and abstract review and were further consulted during the full text eligibility assessment. All co-authors reviewed and approved the final list of included articles. The complete manuscripts of the remaining articles were then assessed against the eligibility criteria. The articles that remained eligible were used in this scoping review.

Once all eligible articles were identified, the final charting table described each article with its citation (author, title, journal, and publication year), geographic location, population, study name, mHealth tool, phase (study type), sample size, behaviors targeted, study outcomes, and findings. Once all data were charted, an overview of the studies and characteristics was synthesized in writing, grouped by the type of mHealth tool used (SMS text message, app, telehealth, video, and other digital tools).

## Results

### Article Selection

**Figure 1:**
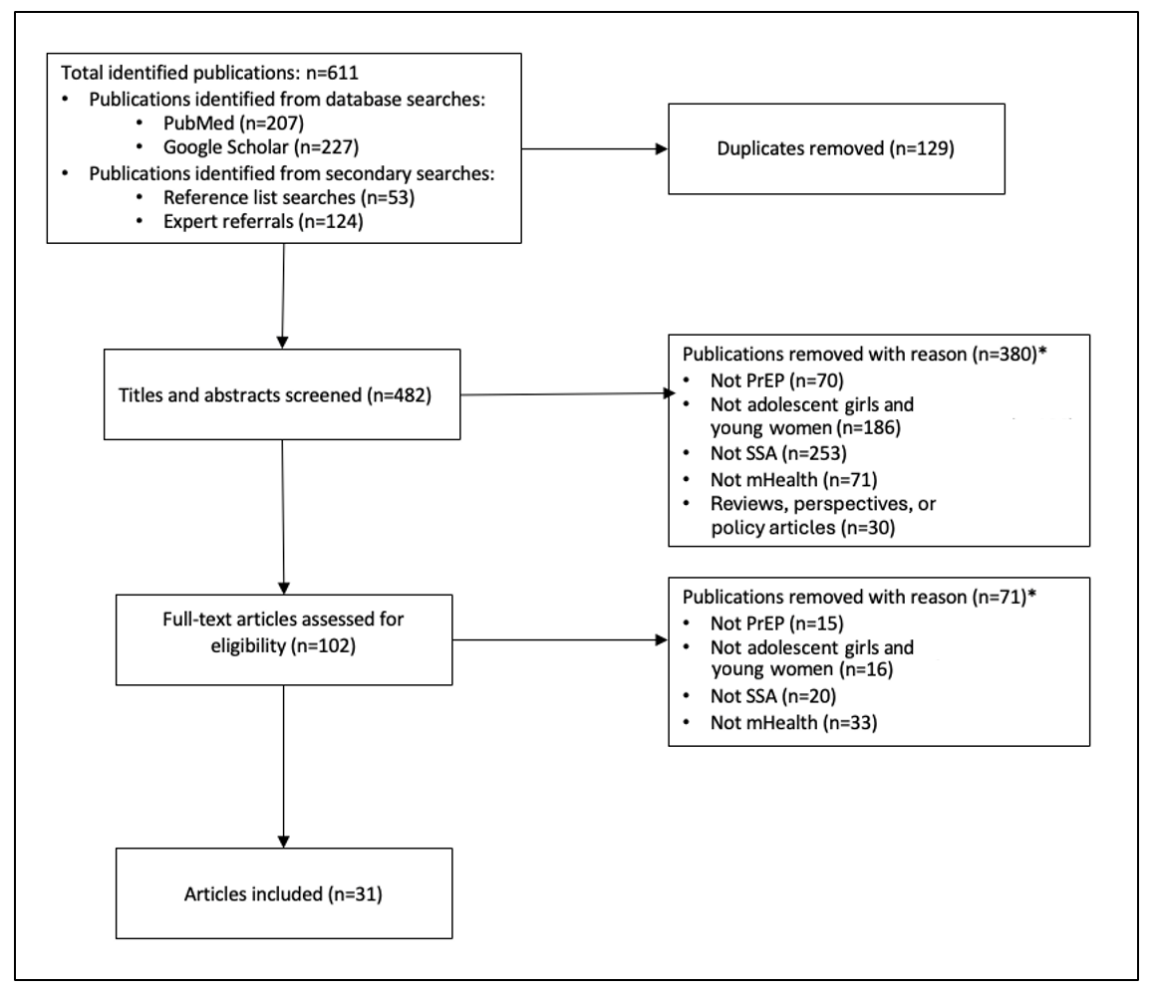
Flow diagram of article inclusion. mHealth: mobile health; PrEP: pre-exposure prophylaxis; SSA: sub-Saharan Africa. *Excluded publications may have been removed for more than one reason.

A total of 611 publications were initially identified through database searches of PubMed and Google Scholar, as well as secondary searches through reference lists and expert referrals. Of these, 129 duplicate records were removed, and the remaining 482 publications underwent title and abstract screening, where 102 articles were selected for full-text screening against the eligibility criteria (Figure 1). Although not mutually exclusive, the most common reasons for exclusion across the 451 excluded articles were not including a study population involving adolescent girls and young women (n=202) and not conducting the study in sub-Saharan Africa (n=273). The remaining 31 articles met the inclusion criteria. They contained information on 21 unique mHealth interventions for PrEP and targeted adolescent girls and young women in sub-Saharan Africa. Several publications used SMS text messages or phone calls for data collection or reminders, as part of the standard of care; however, there was no theory of change associated with PrEP for these interventions, and they were therefore not included in this review.

### Publication Characteristics

Table 1 presents the descriptive characteristics of the included studies. Of the 31 publications, 11 (35%) evaluated outcomes, with study designs that included RCT, pilot study (not powered to assess efficacy), pre/post evaluation, and descriptive study. Moreover, 13 publications (42%) presented formative results, including feasibility, acceptability, qualitative, and modeling studies, and 7 publications (23%) were protocols. For protocol publications, the anticipated characteristics have been included, where appropriate. No publications were published before 2018. Since 2018, there have been at least four publications per year, with 2023 having the highest number of publications (10/31, 32%). Most publications (27/31, 87%) reported sample sizes, which ranged from 50 to 7593; however, samples were generally small, with only 6 out of the 31 samples (19%) being above 500. There were 21 different interventions represented by the 31 publications, with 5 publications resulting from the MPYA study, and 2 each investigating the 3Ps for Prevention study; Determined, Resilient, Empowered, AIDS-free and Safe (DREAMS) study; HPTN 082 study; Jichunge study; mWACh study; and Safer Conception Intervention for Partners (SCIP) study.

**Table 1:** Publication characteristics.

Article, author and year	Study name	Study type	Country	Population	Sample size, n	mHealth^a^ tool
Celum et al [29], 2020	3Ps for Prevention	Outcomes	South Africa	Adolescent girls and young women	200	Telehealth
Morton et al [30], 2020	3Ps for Prevention	Formative	South Africa	Adolescent girls and young women	320	Video
Hartmann et al [31], 2023	CHARISMA^b^	Formative	South Africa	Includes adolescent girls and young women	159	Website
Medina-Marino et al [32], 2021	Community PrEP^c^	Protocol	South Africa	Adolescent girls and young women	480	SMS text message, app
Lavoie et al [33], 2023	DREAMS^d^	Formative	Botswana	Adolescent girls and young women	131	App
Barnabee et al [34], 2023	DREAMS	Outcomes	Namibia	Adolescent girls and young women	7593	SMS text message, telehealth
Kiptinness et al [35], 2023	ePrEP	Protocol	Kenya	Includes adolescent girls and young women	500	Website
Celum et al [12], 2021	HPTN 082	Outcomes	South Africa, Zimbabwe	Adolescent girls and young women	451	SMS text message
Velloza et al [36], 2020	HPTN 082	Formative	South Africa, Zimbabwe	Adolescent girls and young women	67	SMS text message
Mauka et al [37], 2021	Jichunge	Formative	Tanzania	Female sex workers	60	App
Mbotwa et al [38], 2022	Jichunge	Formative	Tanzania	Female sex workers	470	App
Kreniske et al [39], 2023	Kirabo	Protocol	Uganda	Includes adolescent girls and young women	200	SMS text message
Busang et al [40], 2023	Let’s Talk	Protocol	South Africa	Includes adolescent girls and young women	2800	SMS text message, telehealth
Moorhouse et al [41], 2019	Manicaland	Formative	Zimbabwe	Adolescent girls and young women	—^e^	SMS text message
Hanass-Hancock et al [42], 2023	Masibambane–Ladies Chat	Formative	South Africa	Adolescent girls and young women	100	App
Ngure et al [43], 2021	MPYA^f^	Formative	Kenya	Adolescent girls and young women	50	SMS text message
Haberer et al [44], 2021	MPYA	Outcomes	Kenya	Adolescent girls and young women	348	SMS text message
Haberer et al [45], 2021	MPYA	Outcomes	Kenya	Adolescent girls and young women	348	SMS text message
Haberer et al [16], 2022	MPYA	Outcomes	Kenya	Adolescent girls and young women	348	SMS text message
Ogello et al [46], 2023	MPYA	Formative	Kenya	Adolescent girls and young women	54	SMS text message
Cassidy et al [47], 2022	MSF^g^ PrEP Pilot	Outcomes	South Africa	Adolescent girls and young women	164	App
Mogaka et al [48], 2023	mWACh^h^	Protocol	Kenya	Includes adolescent girls and young women	—	SMS text message
Pintye et al [49], 2020	mWACh	Outcomes	Kenya	Adolescent girls and young women	190	SMS text message
Celum et al [50], 2023	MyPrEP	Outcomes	South Africa	Adolescent girls and young women	386	Website
Thomas et al [51], 2019	NCT0356557	Protocol	Zimbabwe	Adolescent girls and young women	1200	App
Celum et al [52], 2022	POWER^i^	Outcomes	Kenya, South Africa	Adolescent girls and young women	2550	SMS text message, app, telehealth
Schwartz et al [53], 2018	PrEP Communications Accelerator	Formative	Kenya	Adolescent girls and young women	530	Website
Velloza et al [54], 2022	PrEP SMART^j^	Protocol	South Africa	Adolescent girls and young women	500	SMS text message, app
Heffron et al [55], 2019	SCIP^k^	Formative	Kenya	HSCs^l^	74 couples	SMS text message
Velloza et al [56], 2019	SCIP	Formative	Kenya	HSCs	74 couples	SMS text message, app
Matambanadzo et al [57], 2021	Sisters with a Voice	Outcomes	Zimbabwe	Female sex workers	—	WhatsApp, website

^a^mHealth: mobile health.

^b^CHARISMA: Community Health Clinic Model for Agency in Relationships and Safer Microbicide Adherence.

^c^PrEP: pre-exposure prophylaxis.

^d^DREAMS: Determined, Resilient, Empowered, AIDS-free and Safe.

^e^Not applicable.

^f^MPYA: Monitoring PrEP among Young Adult Women.

^g^MSF: Médecins Sans Frontières.

^h^mWACh: Mobile Solutions for Women’s and Children’s Health.

^i^POWER: Prevention Options for Women Evaluation Research.

^j^SMART: Sequential Multiple Assignment Randomized Trial.

^k^SCIP: Safer Conception Intervention for Partners.

^l^HSC: heterosexual couples.

Regarding mHealth tools, the studies employed a total of 28 mHealth interventions, as 7 studies investigated more than one mHealth tool (Community PrEP [32], POWER [52], PrEP SMART [54], SCIP [55,56], Sisters with a Voice [57], 3Ps for Prevention [29,30], and DREAMS [33,34]). Of the 28 intervention tools, 11 (39%) included SMS text message tools, 9 (32%) included apps, 3 (11%) included telehealth, 4 (14%) included websites, and 1 (4%) included videos delivered through mobile devices.

Of the 21 unique intervention studies, 19 involved individual locations across 7 countries and 2 (HPTN 082 and POWER) involved locations in 2 countries. Of the 23 locations, 9 (39%) were in South Africa, 6 (26%) in Kenya, 4 (17%) in Zimbabwe, and 1 (4%) each in Uganda, Botswana, Namibia, and Tanzania. Adolescent girls and young women were the sole study population in 62% (13/21) of samples and were part of the population in 24% (5/21) of samples, while female sex workers and heterosexual serodiscordant couples (both populations include adolescent girls and young women) were the sole study population in 10% (2/21) of samples and were part of the population in 5% (1/21) of samples. The key findings for each type of mHealth tool are presented below. When an intervention employed more than one tool, the mHealth intervention component is discussed in its respective section.

### SMS Text Message

Eighteen publications on SMS text message tools for PrEP in adolescent girls and young women reported findings from 11 different studies (Multimedia Appendix 1). The MPYA study involved an RCT of 348 adolescent girls and young women, where the intervention group received personalized daily 1-way SMS text message reminders for PrEP via an automated platform and the control group received no reminders [16,44]. Participants could opt for SMS text message reminders to be sent only after missed doses and could personalize the messages, choose message content, and update these choices during any clinic visit. In this study, adherence was measured via pharmacy refills and a real-time electronic monitor (Wisepill). SMS text message reminders were not associated with greater PrEP adherence over 24 months (47/173, 27.0% with SMS; 46/174, 26.6% without SMS) [16,44]. Among a subset of MPYA participants, in-depth exit interviews revealed that SMS text message reminders were initially highly acceptable, as they were perceived as helping form a daily adherence habit; however, over time, participants reported growing concerns about privacy and SMS text message fatigue [46]. Other barriers included phone loss, poor network connectivity, and lack of electricity [46]. Another qualitative study assessed the perceived risk of HIV and sexual behaviors among MPYA participants [43]. This publication suggested that HIV risk perception was dynamic among participants and may have influenced their PrEP use over time; however, it did not report additional findings related to participants’ receipt of SMS text messages in the study [43].

The mWACh study in Kenya was a 2-group, quasiexperimental, pre-post evaluation of a 2-way SMS text message intervention with 190 pregnant adolescent girls and young women [49]. The intervention was adapted from the existing mWACh 2-way SMS text message platform by introducing theory-based, PrEP-tailored weekly messages, which participants could respond to, and participants could ask questions, which would be replied to by nurses [49]. Compared to participants who did not receive the intervention, participants who received the SMS text message intervention were more likely to return for their first PrEP follow-up (101/190, 53.2% vs 67/166, 40.4%) and to have high PrEP adherence (<1 missed pill per week) at 1 month after starting PrEP and receiving the SMS text messages (82/190, 43.2% vs 37/166, 22.3%) [49]. Promising results from the pre-post evaluation led to an RCT study to evaluate the mWACh SMS text message intervention. Enrollment for the RCT began in March 2022, and the results are expected in 2025 [48].

In Namibia, 7593 adolescent girls and young women participated in DREAMS and received PrEP services via 3 models, based on geographic location: *community-concierge*, *community-fixed*, and *hybrid community-clinic* models [34]. All 3 models had the same PrEP education, screening, and initiation, but differed in follow-up, monitoring, and support. In the *hybrid community-clinic* model, adolescent girls and young women were referred to their public health facility for PrEP refills and clinical monitoring, while the *community-fixed* model had nurses provide refills, monitoring, and follow-up services via scheduled visits at fixed community-based locations. The *community-concierge* model provided refills and monitoring at community-based locations, tailored to participants’ real-time preferences, and offered mHealth support that included visit and refill reminders via SMS text messages and a nurse-managed phone that adolescent girls and young women could message with questions. During the study, 2047 adolescent girls and young women were expected to receive a PrEP refill, but only 254 out of 2047 (12.4%) persisted with PrEP for at least 1 month. Adolescent girls and young women in the *community-concierge* model were 8.7 times (95% CI 5.44-13.9) more likely to persist compared to the *hybrid community-clinic* model; however, there were no specific findings related to the uptake or effect of the SMS text message components of the *community-concierge* model [34].

The SCIP study with 74 heterosexual serodiscordant couples (148 participants) from Kenya evaluated a tablet-based counseling app that provided HIV prevention counseling for the HIV-negative partner, adherence counseling for the partner living with HIV, and information about peak fertility timing [55]. Content for the app was informed by results from an SMS text message survey that collected fertility, PrEP, and ART adherence data. An uptake and usability pilot found that 78.0% (13,181/16,905) of daily surveys were fully answered, and 81.0% (68/84) of HIV negative partners had high PrEP adherence in the months preceding pregnancy [55]. An accompanying feasibility and acceptability study confirmed high SMS text message response rates; however, there was no evaluation of the SMS text message survey’s ability to influence PrEP, and findings from the tablet-based counseling app [56] will be discussed in the App section.

For HPTN 082 in South Africa and Zimbabwe, the intervention being tested involved enhanced adherence support with feedback on tenofovir levels from dried blood spots taken twice during the first 3 months on PrEP. The control condition included 2-way SMS text messages as part of the standard adherence support, along with in-person counseling and in-person peer adherence clubs. From the RCT with 451 adolescent girls and young women [12], at month 6, there was no statistical difference in PrEP adherence between the intervention and control arms (38/179, 21.2% vs 40/184, 21.7%; *P*=.76), and the authors speculated that this may have been due to a strong effect from the standard support measures (including SMS text messaging), which may have been comparable to the benefits of drug-level feedback. During the qualitative study [36], adolescent girls and young women identified stigma and disclosure as initial concerns, but peer adherence clubs empowered many to disclose PrEP and open up about stigma. The adherence clubs, however, were not feasible for all to attend, so participants suggested offering adherence club content digitally (via WhatsApp) to reduce barriers associated with in-person clubs.

The POWER study was a prospective implementation study of PrEP uptake and continuation among 2550 adolescent girls and young women from Kenya and South Africa [52]. Participants received HIV prevention counseling and were offered PrEP. Among the cohort, 94.0% (2397/2550) initiated PrEP, with 31.3% (749/2397) refilling PrEP at the 1-month follow-up. SMS text messages, WhatsApp messages, and phone calls were used to remind participants of quarterly follow-up visits for up to 36 months. Among the participants who were reachable for the 6-month follow-up, 19.8% (128/646) had continued PrEP use, and the authors suggested that the lack of travel and study participation reimbursement may have contributed to this decline [52]. No evaluation of mHealth components was reported.

Upcoming research includes the Manicaland RCT study in Zimbabwe, a 6-month intervention delivering 1-way, tailored, personalized SMS text messages that aim to “nudge” participants toward PrEP adherence by increasing adherence self-efficacy and awareness of HIV risk [41]. Another protocol describes an ongoing study, PrEP SMART, with adolescent girls and young women in South Africa that initially randomizes participants to 1 of 2 mHealth-enhanced interventions to improve PrEP adherence: standard-of-care PrEP counseling complemented by weekly 2-way SMS text messages (modeled after HPTN 082) and standard-of-care PrEP counseling accompanied by WhatsApp adherence groups (described below) [54]. At month 3, nonresponders will continue to receive their original intervention plus either drug-level feedback counseling or intensive problem-solving counseling (no additional mHealth components at rerandomization) [54]. Another protocol describes the Community PrEP Study, which is ongoing in Eastern Cape, South Africa, with a 3-armed RCT comparing the effectiveness for PrEP adherence of the following: in-person health clubs plus WhatsApp adherence groups, one-to-one in-person counseling sessions, and a control condition that includes SMS text message and phone call reminders for study visits [32].

Another protocol is for the Kirabo study in Uganda, where the information, motivation, and behavior model is used to develop a 2-way SMS intervention to increase HIV testing and link users to mental health counseling [39]. The RCT, with a target of 200 youth (including adolescent girls and young women), began in August 2023, and a secondary outcome of the study is the intervention’s impact on PrEP adoption [39]. The final protocol is for Let’s Talk in South Africa, which is a 2-armed, stepped-wedge trial involving 2800 participants, with a primary outcome of PrEP uptake for HIV-negative participants [40]. The control arm will receive the current standard of care, while the intervention arm will receive social mobilization and tailored support, provided by peer navigators and adolescent and young adult–friendly nurse-led mobile clinics. All participants who start PrEP in the intervention cluster are offered peer-navigator support as well as neutral text message reminders. The 45-month study is ongoing, and data collection is expected to be complete in 2024 [40].

### App

Twelve publications described 9 different app-based interventions across 10 different studies, including 2 publications on Jichunge, a smartphone app for iOS and Android aimed at increasing PrEP adherence among female sex workers and men who have sex with men in Tanzania (Multimedia Appendix 2). One publication described the participatory design approach to the Jichunge app [37]. An information system research framework was employed so that participants from the target demographics (including 15 female sex workers in the design phase and 20 female sex workers in the testing phase) could inform app content and the accompanying web-based administrative portal. After the design and testing cycles, the final prototype included the following functionalities to promote PrEP adherence: gamification (awarding levels and points for completing tasks in the app), drug registration on the in-app medication tracker, medication time reminders, notifications, communication with peers, communication with health care workers, discussion forum, educational material, and quizzes [37]. Jichunge was then evaluated in a quasiexperimental study with 470 female sex workers, where end-user data from the app and qualitative interviews were used to evaluate “optimal” app use (defined as using 3 app functions in the first 30 days) [38]. Overall, 46.0% (216/470) of women met this definition of optimal app use, with substantially higher use among women over 35 years of age and those with prior social media experience [38]. The effectiveness of the app on adherence or health outcomes was not assessed.

The Médecins Sans Frontières (MSF) PrEP Pilot in South Africa was an observational cohort of 224 adolescent girls and young women, which investigated PrEP uptake and adherence with an intervention that was guided by motivational interviews and the “stages of change” theory [47]. The intervention included in-person counseling sessions (covering PrEP use, HIV risk assessment and reduction, and sexual and reproductive health information); “PrEP Diva” gatherings, which replaced traditional “sexual and reproductive health clubs;” and a WhatsApp group where participants could engage with study staff and each other via text messages. PrEP was initiated by 73.2% (164/224) of participants. High adherence was defined as ≥700 fmol/punch (the equivalent of taking at least four pills per week), and at the 1-month follow-up, 17.7% (29/164) had high adherence; however, this dropped to 12.2% (20/164) at month 18. Forgetting to take or travel with pills was the main barrier to adherence, and over the final 12 months, encouragement from others declined as a facilitator of adherence [47].

Sisters with a Voice was an observational study involving 6539 female sex workers in Zimbabwe that investigated PrEP uptake before and after the scale-up of differentiated service delivery (DSD) models in response to COVID-19 lockdowns [57]. The DSD models included digital components like online appointment scheduling, WhatsApp, and telehealth, as well as nondigital components like peer demand generation to increase uptake, community-based delivery, and multi-month medication dispensing. For WhatsApp, this platform allowed continuous virtual engagement from peer educators, which enabled users to report adverse effects and receive adherence support. From January to March 2020, there was no DSD, and PrEP uptake rates were less than 25.0% (315 initiations or fewer out of 1260) per month. DSD was scaled up in April 2020, and in May 2020, PrEP uptake began increasing each month, peaking at 51.0% (1360 initiations out of 2667) in September 2020. PrEP uptake data were not disaggregated by use of or exposure to the various DSD components, limiting the ability to draw conclusions regarding the impact of the digital health tools [57].

The development and pilot testing of Masibambane (Let us Unite)–Ladies Chat was based on the information, motivation, and behavioral skills model; social learning theory; and gender theory to promote PrEP uptake and knowledge among adolescent girls and young women in South Africa. A publication describes the formative development and pilot testing of the intervention, which was designed based on 9 existing group-based activities (traditionally performed in person) and adapted to be delivered using a hybrid approach primarily on WhatsApp, which included asynchronous chat and live sessions [42]. Lessons learned from the pilot included a need to work around the impact of technology access (availability of free data at night and electricity outages [“load shedding”]), enhancing participant engagement through staff modeling engagement and by using mixed communication styles (verbal, written, and emoji), and introducing breaks during long sessions. No further evaluation or outcome information was provided.

While the abovementioned DREAMS program in Namibia evaluated DSD models, the DREAMS Botswana program was a separate, formative study that assessed the feasibility and acceptability of providing peer-delivered mHealth interventions (eg, WhatsApp groups) to support PrEP use among 131 adolescent girls and young women [33]. Feasibility was high, as 89.3% (117/131) of participants owned a mobile phone and 96.9% (127/131) used WhatsApp in the last year. Moreover, 84.7% (111/131) stated they would be interested in a peer-delivered mHealth intervention, 78.6% (103/131) preferred SMS text message or phone call reminders for PrEP refills, and the average acceptability of the intervention measure was 3.8 out of 5 [33].

The SCIP study, which has been introduced above, used a tablet-based Android app for counseling on fertility timing, PrEP, and ART adherence to complement the SMS text message survey [55]. The feasibility and acceptability study confirmed that the SMS text message and MEMS Cap data could be successfully integrated into the tablet-based app, with SMS text message data integrated via an mSurvey application programming interface (Kenyan mobile data collection company) and the MEMS Cap data downloaded directly to the tablet during clinic visits. The app was not available to participants on their personal smartphones; however, the tablet-based app was available during clinic visits to complement in-person counseling on fertility timing, PrEP, and ART adherence. No evaluation was presented of the app’s ability to influence PrEP adherence or maintenance [56].

In the POWER study, which has been introduced above, WhatsApp was offered as a platform to remind participants of quarterly follow-up visits for up to 36 months, similar to the SMS text message intervention. No further description of the WhatsApp intervention or evaluation of the mHealth components was reported [52].

The remaining 3 studies on apps have been published as study protocols for RCTs (no results available to date). The PrEP SMART study, which has been described above, includes WhatsApp adherence groups with 25-50 participants each to facilitate social and emotional support [54]. The adherence groups are facilitated by a staff member and a PrEP-experienced peer (“PrEP ambassador”) and include educational and fun activities. The Community PrEP Study, which has been described above, includes 1 intervention arm that consists of health clubs with 8 to 10 participants who attend in-person group counseling sessions and have access to a club-specific WhatsApp group [32]. The third protocol is for an RCT in Zimbabwe with 1200 adolescent girls and young women (NCT03565575) from July 2018 through August 2020 [51]. This pilot RCT evaluated the ability of interactive digital tablet-based quizzes and counseling sessions to increase PrEP uptake compared to a control arm with no intervention. Though data collection ended in 2020, no published findings were identified during our literature search, and no information was obtained after attempting to contact the corresponding author.

### Telehealth

Three studies included telehealth-based intervention components (Multimedia Appendix 3). The Sisters with a Voice observational study of DSD models to increase PrEP uptake, presented in the App section, had additional components, including telehealth and websites for appointment scheduling. The telehealth scale-up included the ability for participants to report adverse results by phone and to receive telehealth follow-ups for side effects and adherence counseling. As noted above, while the overall intervention package increased PrEP uptake, the effectiveness of or exposure to individual components was not evaluated [57].

As described in the SMS Text Message section, participants in DREAMS Namibia received PrEP services via 3 models: *community-concierge*, *community-fixed*, and *hybrid community-clinic* models. In the *community-concierge* model, which was 8.7 times more likely to experience PrEP persistence (than the hybrid model), the mHealth components (visit and refill reminders, and a nurse-managed phone with questions) could also be accessed by phone; however, no specific findings were reported related to the uptake or effect of the telehealth components [34]. The Let’s Talk protocol, which has been described above, included additional mHealth components beyond the neutral text reminders [40]. Participants in the peer-navigator intervention group were also offered the choice to receive reminders by phone, instead of text, and a nurse-managed phone hotline was available 24/7 for participants seeking psychological support or clinical guidance. Three additional studies that had other mHealth components (3Ps for Prevention [29], POWER [52], and Community PrEP Study [32]) also used phone calls for clinic or study visit reminders; however, this activity was not evaluated as a PrEP-focused mHealth intervention.

### Website

Four studies investigated a website or mobisite as part of the intervention, including MyPrEP, a client-facing mobisite with a decision support tool designed to improve users’ risk perceptions, PrEP knowledge, informed decision-making, and motivation, in order to facilitate PrEP delivery (Multimedia Appendix 3). The study involved an RCT conducted in South Africa with 386 adolescent girls and young women, where the control group was directed to a general health website, while the intervention group was directed to the MyPrEP decision support tool mobisite. The decision support tool, which was originally developed for an electronic tablet and then reformatted for smartphone web access, presented available HIV prevention options in an impartial manner, supporting users to make informed decisions, including whether to initiate PrEP. PrEP initiation was similar across both groups, with 97.1% (167/172) initiating in the MyPrEP group and 93.9% (170/181) in the control group (odds ratio 1.79, 95% CI 0.79-1.53); however, PrEP persistence at 1 month (determined by a documented PrEP refill) was 19.2% (32/167) in the MyPrEP group and only 10.0% (17/170) in the control group (odds ratio 1.97, 95% CI 1.08-3.69) [50].

One formative study, PrEP Communications Accelerator, described the development of a website [58] to help programs generate demand for PrEP in sub-Saharan Africa by providing a customized communications plan for targeted audiences (including adolescent girls and young women and female sex workers) and according to the health care setting. Each tailored demand generation strategy suggested evidence-based media channels and communication tactics, but the outputs have not yet been evaluated for usability, acceptability, or effectiveness [53].

Another formative study investigated CHARISMA (Community Health Clinic Model for Agency in Relationships and Safer Microbicide Adherence), a mobisite designed to mitigate relationship barriers to PrEP, like intimate partner violence [31]. By using human-centered design and workshops, the mobisite was adapted from existing counselor-administered HIV prevention and relationship counseling interventions. The mobisite was further refined by beta-testing with 81 women, followed by a real-world feasibility and acceptability pilot with 159 women. In the pilot test, adolescent girls and young women rated the website 4.5 stars out of 5, found the website useful (4.5 out of 5) and safe (4.5 out of 5), and had minimal confidentiality concerns (1.8 out of 5). Based on these findings, the authors suggested that future research should evaluate health impacts, including PrEP uptake and continuation; however, no supplementary protocols were identified [31].

The ePrEP study involves a protocol for an upcoming single-arm pilot in Kenya that will deliver PrEP via a private online pharmacy retailer, MYDAWA [35]. Potential participants can order an HIV self-test from MYDAWA (250 KES [approximately US $2]) and have it delivered to a location of their choice. They can then upload a picture of their self-test results and attend a telemedicine consult with a MYDAWA clinician to screen and receive counseling for PrEP. Eligible participants will be prescribed a free 30-day PrEP supply, and it will be delivered for 99 KES (approximately US $1). The study will measure PrEP initiation and continuation, postexposure prophylaxis-to-PrEP transition, and implementation outcomes like feasibility, acceptability, and costs. The study is anticipating at least 500 participants over 18 months; however, no expected date of completion has been provided [35].

### Video

An earlier phase of the previously described 3Ps for Prevention study investigated demand generation, which included a qualitative component that examined video and information brochures [30]. A 90-second video was cast with local youth and shot by a professional filmmaker, based on a script created by a marketing firm that conducted focus group discussions and interviews with local adolescent girls and young women on PrEP. Participants were recruited in their own homes by study staff going door-to-door and were shown the video if they agreed to participate. A total of 320 adolescent girls and young women watched the PrEP demand-generation video, and among 300 replies, most (220/300, 73.3%) said they wanted to learn more about PrEP after watching the video. However, without a control group that did not watch the video, this study design does not contextualize the results. PrEP interest was found to be associated with having sex with a primary partner in the last 30 days and being in a sexual partnership for over 6 months [30] (Multimedia Appendix 3).

### Summary of Key Findings

Of the 18 publications on SMS text messages, there were only 2 impact evaluations on the ability of SMS text message interventions to increase PrEP adherence, and they presented inconsistent findings. The mWACh 2-group, quasiexperimental, pre-post evaluation found that SMS text message interventions increased high PrEP adherence after 1 month [49], while the MPYA RCT found that SMS text message reminders did not increase PrEP adherence over the control condition after 24 months [16,44]. A third descriptive study, DREAMS Namibia, evaluated 3 different service models and found that the *community-concierge* model, which included SMS text message reminders and a nurse-operated phone line that could receive SMS text message questions, had 8.7 times higher PrEP persistence at 1 month than the hybrid model [34]. User preference for SMS text messages was noted in the 2 Kenyan studies that reported high SMS survey completion rates; however, there were 2 studies that reported low completion rates. Among the studies that reported high completion rates, 2 reported high SMS text message acceptability and 1 indicated that SMS text message reminders might be helpful in forming daily adherence habits. The anonymity of SMS text messages may reduce social desirability bias, and SMS text messaging as a data collection tool introduces the ability to automate data cleaning and analysis. Three studies reported barriers to SMS text messaging, which included privacy, SMS text message fatigue, and lack of a phone, network, or electricity (Table 2).

For apps, there were no explicit impact evaluations; however, WhatsApp-based interventions were associated with high PrEP uptake in the MSF PrEP pilot [47], and WhatsApp was included as one of the interventions that was scaled up in the Sisters with a Voice study that doubled PrEP uptake in 5 months [57]. In 1 study, 96.9% (127/131) of participants had used WhatsApp in the past year [33], and WhatsApp was also identified as the most accessible platform in another study [42] and suggested as a platform for digital adherence clubs in another, although with some privacy concerns [36]. Although WhatsApp can be used as a text messaging service similar to SMS text messaging, it operates on data, not airtime, which makes it more affordable. Furthermore, it has strong privacy protection through encryption and offers the ability to participate in group chats. Some participants suggested a hybrid approach with asynchronous chat and live WhatsApp sessions that used mixed communication styles (verbal, written, and emojis). The main barriers to app use were data costs and electricity outages.

This review identified a small subset of literature regarding telehealth interventions, websites, and the use of videos to support PrEP for adolescent girls and young women in sub-Saharan Africa. Outside of the forthcoming ePrEP and Let’s Talk studies, the data are very limited. Telehealth (for appointment scheduling, reporting results, and follow-up consultation) was one of the interventions that was scaled-up in the Sisters with a Voice study and was a component of the *community-concierge* model (phone reminders and a nurse-operated phone line that participants could call with questions or concerns) of the DREAMS Namibia study; however, the effects of the individual mHealth components were not presented in either of these studies. Phone calls were also used in several studies as part of the standard of care for appointment reminders and data collection; however, these were not hypothesized or evaluated as interventions to improve PrEP use. Participants reported barriers associated with phone ownership and data costs.

The MyPrEP RCT found that participants who used the DCT mobisite, designed to improve risk perception and support informed decision making, were twice as likely to persist with PrEP at 1 month than the control group. No other studies evaluated the effectiveness of websites; however, the CHARISMA study did show that a mobisite to mitigate relationship barriers associated with PrEP use had high feasibility and acceptability among adolescent girls and young women. For videos, there was no empirical evidence to support their effectiveness at changing PrEP uptake; however, 73% of participants who watched the 3Ps for Prevention video requested more PrEP information after viewing the video.

**Table 2:** Summary of key findings.

mHealth^a^ intervention	Facilitators	Barriers	Evidence for mHealth	Evidence against mHealth^b^
SMS text message	SMS text message reminders were initially highly acceptable and helped form a daily adherence habit (MPYA^c^) [16,44] SMS text message surveys had a high completion rate (MPYA and SCIP^d^) [16,44]	Privacy, SMS text message fatigue, phone loss, poor network connectivity, and lack of electricity (MPYA) [16,44]	Pre-post evaluation found that the 2-way SMS text message intervention group was more likely to continue PrEP^e^ than the group without SMS text message (mWACh^f^) [49] Descriptive study found that a model including SMS text message reminders and asking nurses questions via SMS text messages had an 8.7× higher PrEP persistence at 1 month (DREAMS^g^ Namibia) [34]	RCT^h^ found that 1-way SMS text message reminders were not effective in promoting PrEP adherence (MPYA) [16,44]
App	Digital adherence clubs (WhatsApp) were suggested to reduce barriers of in-person clubs (HPTN 082) [36] WhatsApp as the most accessible platform (Masibambane–Ladies Chat) [42] 89.3% (117/131) owned a smartphone, 96.9% (127/131) had used WhatsApp in the last year, and 84.7% (111/131) were interested in peer-delivered mHealth (DREAMS Botswana) [33] SMS text message and MEMS Cap data integrated into a tablet-based app (SCIP) [55]	Must work around the impact of technology access like availability of free data at night and electricity outages (load shedding) (Masibambane–Ladies Chat) [42]	WhatsApp included in scaled-up DSD^i^ that doubled PrEP uptake in 5 months (Sisters with a Voice) [57] WhatsApp groups associated with high PrEP uptake of 73.2% (164/224) (MSF^j^ PrEP pilot) [47] Optimal app use was 46.0% (216/470) in a pilot (Jichunga) [38]	—^k^
Telehealth	Phone calls for data collection (3Ps for Prevention study) [29]	—	Telehealth included in scaled-up DSD that doubled PrEP uptake in 5 months (Sisters with a Voice) [57] Descriptive study found that a model including phone reminders and asking nurses questions via phone had an 8.7× higher PrEP persistence at 1 month (DREAMS Namibia) [34]	—
Website	Hybrid approach with a chat box and live sessions preferred (Masibambane–Ladies Chat) [42] Mixed communication styles (verbal, written, and emoji) preferred (Masibambane–Ladies Chat) [42] Pilot study of mobisite to mitigate relationship barriers to PrEP showed high feasibility and acceptability (CHARISMA^l^) [31]	—	RCT found that the mobisite decision support tool group was twice as likely to persist with PrEP at 1 month than the control group (MyPrEP) [50]	—
Video	—	—	After watching a video, 73.3% (220/300) wanted more PrEP info (3Ps for Prevention study) [29]	—

^a^mHealth: mobile health.

^b^No clear controlled evidence against mHealth was found in the search.

^c^MPYA: Monitoring PrEP among Young Adult Women.

^d^SCIP: Safer Conception Intervention for Partners.

^e^PrEP: pre-exposure prophylaxis.

^f^mWACh: Mobile Solutions for Women’s and Children’s Health.

^g^DREAMS: Determined, Resilient, Empowered, AIDS-free, Mentored and Safe.

^h^RCT: randomized controlled trial.

^i^DSD: differentiated service delivery.

^j^MSF: Médecins Sans Frontières.

^k^Not available or not applicable.

^l^CHARISMA: Community Health Clinic Model for Agency in Relationships and Safer Microbicide Adherence.

## Discussion

### Overview

This scoping review found few robust evaluations of mHealth tools that successfully supported PrEP use (uptake, adherence, and persistence) among adolescent girls and young women in sub-Saharan Africa. While previous reviews mainly synthesized information on SMS text message interventions across 6 studies (HPTN 082, 3Ps for Prevention, POWER, PrEP SMART, MPYA, and mWACh) [18,19,25], this review expanded the sample to 21 studies, which included apps, telehealth, videos, websites, and additional SMS text message interventions. The studies presented in this review showed generally high usability and acceptability of mHealth interventions. Additional rigorous study designs and evaluations are needed to measure engagement with mHealth interventions and assess the effectiveness of mHealth interventions to increase PrEP uptake, adherence, and sustained use over time.

### Use of mHealth Tools for Research, Clinical Care, and Program Implementation

Despite the limited conclusive evidence from intervention trials supporting mHealth interventions for PrEP adherence, prevention programs for adolescent girls and young women in sub-Saharan Africa are still adopting and benefiting from these interventions to support program implementation. Numerous studies not included in this review demonstrated the feasibility and acceptability of mHealth tools for the collection of PrEP adherence and sexual behavior data among adolescent girls and young women in these settings [29,55,59-62]. In some studies, mHealth tools were already employed as part of the standard of care or DSD, including 2-way SMS text message adherence and appointment reminders, online appointment scheduling, and telehealth options [12,29,32,57].

In 2023, the World Bank published *Framework for Economic Evaluation of Digital Health Interventions*, which recommends that economic evaluations of digital health interventions include an impact inventory to capture secondary benefits in addition to their anticipated health outcomes [63]. These benefits can include improved user experience, improved health care worker experience, improvements to the health system, value of data generated, and enhanced interoperability across systems. mHealth interventions from this review revealed a number of these secondary benefits that could be captured in an impact inventory, such as high usability and acceptance, the ability to capture data with less social desirability bias [61], and integration into other systems [56].

### Adapting Promising mHealth Approaches Across Geographic Settings and Populations

During the literature search, only 31 publications met the inclusion criteria, while 202 were excluded for predominantly investigating men who have sex with men in various countries, including some in sub-Saharan Africa. This uneven distribution mirrors the general promotion of PrEP in the global North and among men who have sex with men [64]. Indeed, prior reviews of mHealth interventions for adolescent girls and young women in LMICs found none conducted outside of sub-Saharan Africa [18,19,25]. Our additional searches identified a number of studies for adolescent girls and young women in the United States and 1 in Brazil; however, almost all reported on formative phases [65-67].

The larger body of published research, including impact evaluations, among sexual and gender minority men has begun to show the effectiveness of mHealth interventions on PrEP for men who have sex with men [20,21]. Lessons learned from these settings could inform mHealth intervention adaptations to accelerate the process from development to implementation. In 2019, an RCT in Chicago, United States showed that a 2-way SMS intervention increased PrEP adherence by 15% over 36 weeks [23], and in 2021, a pilot study in Boston, United States revealed that a culturally and developmentally tailored PrEP adherence app nearly doubled perfect adherence rates after 6 weeks of app use, compared to a control [22]. Although all participants were men who have sex with men, the information, motivation, and behavioral skills theory of behavior change behind the medication reminders and supportive messages designed to improve PrEP knowledge and adherence may also resonate with adolescent girls and young women in sub-Saharan Africa [42]. Tailored mHealth interventions have the potential to be effective across different demographics, especially since both men who have sex with men in the United States and adolescent girls and young women in sub-Saharan Africa have expressed similar barriers to PrEP, including fear of stigma and discrimination, privacy, and long clinic wait times [25,68].

### Considerations for Ongoing Development of mHealth PrEP Tools for Adolescent Girls and Young Women

Mobile penetration continues to rise in sub-Saharan Africa, and delivering information via these platforms may involve lower costs as compared to other modalities like facility-based counseling or mobile outreach clinics. In 2021, there were over 500 million unique mobile subscribers in the region, and that number is expected to reach 613 million by 2025, as an increasing number of tech-savvy youth reach the age where they can begin to purchase mobile subscriptions [69]. The COVID-19 pandemic caused a global shift toward the acceptance of and reliance on digital interventions, including those for health care [70,71]. Furthermore, in 2022, the WHO and UNAIDS released a policy brief titled “Virtual interventions in response to HIV, sexually transmitted infections and viral hepatitis,” which suggested that virtual interventions should be introduced to complement existing processes by streamlining the exchange of information and improving access to care [72].

User preferences from this review include simple wording in a variety of languages as part of a hybrid mHealth approach with an affinity for WhatsApp, which is in line with the incorporation of mHealth tools as a component of decentralized DSD [73]. Implementers must be aware of use barriers, such as SMS text message fatigue, lack of data, privacy concerns, unstable network coverage, and power outages [42,46]. A hybrid approach with curated content may help reduce SMS text message fatigue, while data vouchers can be offered to supplement mHealth use. Furthermore, the widespread popularity of WhatsApp likely reflects the many network providers in sub-Saharan Africa that offer low-cost or free unlimited WhatsApp use with most plans, and the built-in encryption may address privacy concerns [74].

### Knowledge Gaps and Future Directions

There are knowledge gaps that still exist, including the lack of well-controlled studies determining the effectiveness of mHealth tools for increasing uptake, adherence, and continuation of oral PrEP regimens in adolescent girls and young women living in sub-Saharan Africa. Additional gaps include the lack of “best practice” approaches to translate evidence-based interventions into mHealth delivery and the inconsistent use of behavioral theory of change frameworks. If future methodologies begin to address these gaps, future results should provide a deeper understanding of how variations in impact may be attributed to differences in the types of mHealth tools used, the content of intervention messages, and the broader implementation science approaches employed. As new models of PrEP delivery, such as next-generation long-acting PrEP [75] and pharmacy distribution [76], expand and the landscape shifts toward decentralized service delivery, mHealth interventions should continue to support their implementation and improve adherence. Future research should ideally be designed with consideration of implementation science methodologies and frameworks to ensure that the effectiveness of mHealth interventions for PrEP adherence is being accurately captured while also measuring economic impact and auxiliary benefits [19,63]. In a recent review of the implementation of mHealth interventions for HIV or sexually transmitted infection prevention in LMICs, researchers applied the taxonomy of implementation outcomes by Proctor et al [77] (acceptability, adoption, appropriateness, feasibility, fidelity, implementation cost, penetration, and sustainability) and found few studies that measured these outcomes with a bias focused on acceptability, appropriateness, and feasibility and limited to no measurement of adoption, fidelity, cost, penetration, and sustainability [78]. Moreover, even fewer studies have used or reported on implementation strategies (eg, audit and feedback, identify and prepare champions, and conduct ongoing training [79]) for enhancing how digital HIV prevention and care interventions are delivered and supported (J Brasileiro, unpublished data, November 2024). Without more systematic application and measurement of implementation strategies and outcomes, the field will continue to struggle in the interpretation of mixed and conflicting results from mHealth PrEP interventions.

Forthcoming interventions and studies should also be cognizant of barriers to mHealth among adolescent girls and young women in sub-Saharan Africa, including privacy, SMS text message fatigue, shared phone ownership, network and electricity interruptions, and data costs. Greater understanding of medical distrust and other perceptions of PrEP among adolescent girls and young women in this context should also be taken into consideration.

### Limitations

This scoping review had several limitations. Although this review focused on adolescent girls and young women, publications on heterosexual couples and female sex workers (adolescent girls and young women make up a significant proportion of each population) were also included, although these populations may have slightly different drivers toward PrEP than adolescent girls and young women independently. Our literature search identified a number of mHealth interventions, such as B-wise [80], a website with PrEP information for adolescent girls and young women in South Africa, and Tutu Tester [81], a mobile clinic that used mHealth to connect with HIV testers in the community [82,83]; however, these interventions were not included, as their impacts from mHealth were not published in peer-reviewed journals or as conference abstracts. A lack of robust evaluation of mHealth interventions reflects the current funding environment in sub-Saharan Africa, which is more focused on implementation and demonstration projects, and the limitation of the field of digital health, with many tools struggling to get past development and pilot testing to large scale trials owing to the nature of the technology sector and incompatible health research funding models [84]. Several studies in this review evaluated the values and preferences of mHealth or used mHealth as part of the implementation strategy (data collection, standard of care, or DSD), and very few studies used impact evaluations like RCTs or pre-post evaluations. Furthermore, most of the studies that evaluated impact were pilot studies or had small samples that had not yet been scaled up. With quasiexperimental designs and underpowered samples, it is unclear whether insignificant findings were due to ineffective mHealth interventions or weak implementation strategies.

### Conclusion

Findings from this scoping review suggest that, to date, there is insufficient evidence supporting the effectiveness of mHealth interventions for increasing PrEP uptake, adherence, and sustained use among adolescent girls and young women in sub-Saharan Africa, despite high usability and acceptability across different platforms. Despite the lack of an evidence base, several studies and interventions have already adopted mHealth tools for data collection or as part of their standard of care, suggesting that they may be providing secondary benefits. Furthermore, digital technologies have become more accessible, and their use by young people has grown exponentially. Implementation science frameworks and methodologies, such as RCTs with statistically significant sample sizes, should be considered in future research to accurately capture the effectiveness of mHealth interventions for behavior changes leading to improved PrEP use. This should allow for the identification and scale-up of impactful interventions while also providing direction as to how these mHealth tools may be tailored to potentially improve different areas of care.

## Appendix

Multimedia Appendix 1 Summary of SMS text message publications.

Multimedia Appendix 2 Summary of app publications.

Multimedia Appendix 3 Summary of other publications.

Multimedia Appendix 4 PRISMA-ScR (Preferred Reporting Items for Systematic Reviews and Meta-Analyses extension for Scoping Reviews) checklist.

## Abbreviations

ART: antiretroviral therapy
CHARISMA: Community Health Clinic Model for Agency in Relationships and Safer Microbicide Adherence
DREAMS: Determined, Resilient, Empowered, AIDS-free and Safe
DSD: differentiated service delivery
LMIC: low- and middle-income country
mHealth: mobile health
MPYA: Monitoring PrEP among Young Adult Women
MSF: Médecins Sans Frontières
mWACh: Mobile Solutions for Women’s and Children’s Health
POWER: Prevention Options for Women Evaluation Research
PrEP: pre-exposure prophylaxis
RCT: randomized controlled trial
SCIP: Safer Conception Intervention for Partners
SMART: Sequential Multiple Assignment Randomized Trial
UNAIDS: Joint United Nations Programme on HIV and AIDS
